# Intermittent theta burst stimulation over the left prefrontal cortex: no additional effect for virtual reality exposure therapy in acrophobia—a randomized trial

**DOI:** 10.1038/s41598-024-80832-1

**Published:** 2024-11-27

**Authors:** L. M. Cybinski, B. Bohmeier, K. Rolle, D. Gromer, T. Raij, F. Gundelach, A. Erhardt-Lehmann, A. Mühlberger, J. Deckert, T. Polak, P. Pauli, M. J. Herrmann

**Affiliations:** 1 Center of Mental Health, Department of Psychiatry, Psychosomatics, and Psychotherapy, University Hospital of Wuerzburg, 97080 Wuerzburg, Germany; 2 Institute of Medical Psychology and Behavioral Neurobiology, University of Tübingen, 72076 Tübingen, Germany; 3 Department of Psychology - Clinical Psychology and Psychotherapy, University of Wuerzburg, 97070 Wuerzburg, Germany; 4 MGH/MIT/HMS Athinoula A. Martinos Center for Biomedical Imaging, Department of Radiology, Massachusetts General Hospital, Charlestown, MA 02129 USA; 5 Department of Translational Research in Psychiatry, Max Planck Institute of Psychiatry, 80804 Munich, Germany; 6 Department of Psychology - Clinical Psychology and Psychotherapy, University of Regensburg, 93053 Regensburg, Germany

**Keywords:** Anxiety, Prefrontal cortex

## Abstract

Anxiety disorders are the most prevalent mental health conditions. Besides psycho-pharmacotherapy, cognitive behavioral therapy with an exposure-based approach is considered the gold standard. However, not all patients benefit from this approach. Here, we aimed to translate laboratory findings on enhanced fear extinction with repetitive transcranial magnetic stimulation (TMS) to the clinic. In this double-blind, randomized, placebo-controlled clinical trial, 76 participants with acrophobia received an activating intermittent theta burst stimulation (iTBS) targeting the left posterior prefrontal cortex immediately before two virtual reality exposure therapy sessions. Phobic symptoms were assessed at baseline, post-intervention, and a 6-month follow-up. Results revealed a significant reduction in phobic symptoms from baseline to post-assessment and follow-up and confirmed the efficacy of virtual reality exposure therapy as a treatment for specific phobias. Interestingly, no additional effect was observed for active iTBS compared to sham iTBS. Our post-hoc analyses argue for an individualized TMS application. Further research is needed to determine optimal TMS parameters and validate these results in clinical trials, accounting for methodological and inter- and intra-individual variability, as well as alternative therapeutic processes.

## Introduction

With a lifetime prevalence of about 17%, anxiety disorders are the most common mental health condition worldwide, with specific phobias representing the largest subgroup, affecting approximately 5% of the global population^1^. Anxiety disorders are characterized by excessive fear and anxiety, leading to behavioral disturbances, significant distress, and impairments in daily functioning^2^. Untreated individuals are at higher risk of experiencing chronic symptoms and developing comorbidities such as other anxiety disorders, depression, and substance abuse, imposing a substantial individual and societal burden^3^. Together with the associated administrative and economic challenges in primary care^4^, there is an urgent need for highly effective treatments for anxiety disorders.

Besides psychopharmacotherapy, cognitive-behavioral therapy (CBT) is currently the most effective treatment for anxiety disorders^5,6^, with exposure-based CBT showing superior effects when compared to CBT without exposure^7^. Along with habituation models^8^, classical fear conditioning and extinction paradigms have been extensively investigated and have emerged as valid translational models for anxiety disorder treatment^9,10^. Enhanced extinction learning, for instance, has been positively associated with CBT outcomes^11^ and is considered a crucial underlying learning process in exposure therapy. This has sparked tremendous interest in neuroscience research to elucidate the underlying neurobiological mechanisms of fear extinction in both healthy individuals and those with anxiety disorders, as well as treatment processes and outcomes. Systematic reviews and meta-analyses have emphasized the critical role of the ventromedial prefrontal cortex (vmPFC) in the recall of extinction learning in healthy humans^12–14^, with top-down regulatory pathways to the amygdala^15^ particularly involved in threat detection and responses^16^. These results are consistent with research conducted in rodents^17–19^. However, patients with anxiety disorders exhibit dysfunctional amygdala-prefrontal connectivity^20,21^ and impaired fear extinction, resulting, for example, in increased fear responses to threat stimuli during extinction compared to controls^22^. In addition to various factors, such as trait anxiety and motivation, that may influence the efficacy of exposure therapy^23^, these findings may explain why approximately 50% of patients with anxiety disorders do not respond significantly to treatment^24^, and one-third experience a return of fear after successful therapy^25^. Consequently, there is a pressing need to optimize therapeutic strategies by, for instance, enhancing fear extinction processes to improve treatment efficacy in anxiety disorders.

Non-invasive brain stimulation (NIBS) techniques are promising new alternative therapeutic approaches and have shown preliminary evidence for enhanced symptom reduction in anxiety disorders, with an overall medium effect size^26^. These techniques are considered safe methods and are associated with increased or reduced excitability, depending on the stimulation protocol^27,28^. NIBS can be applied either online, concurrently with task performance, to modulate ongoing neural processes in a task- and timing-dependent manner, or offline, focusing on longer-lasting neuromodulatory aftereffects through repetitive administration^29^. Among NIBS techniques, transcranial magnetic stimulation (TMS) provides superior spatial and temporal resolution compared to transcranial direct current stimulation (tDCS)^29^. TMS is already recommended for the treatment of treatment-resistant depression^30^ and some other mental disorders. While high-frequency (> 5 Hz) or intermittent theta burst stimulation (iTBS) protocols have been shown to increase excitability, low-frequency (< 1 Hz) or continuous TBS protocols have demonstrated suppressive effects on neuronal excitability^27^.

Given that electrical neuromodulation of the infralimbic cortex (IL; homolog of the vmPFC) has been shown to enhance extinction learning processes in rodents^31–33^, NIBS have been increasingly investigated for translating these findings to humans. The aim is to modulate neurophysiological mechanisms in healthy subjects and those with anxiety disorders^26,34^. However, applying TMS to modulate vmPFC activation is challenging due to its deep cortical location and the superficial nature of TMS as a stimulation technique. A recent study investigated the effects of repetitive TMS (rTMS) on fear extinction processes at a more accessible stimulation target, the left posterior PFC (MNI: − 56, 2, 40)^35^. This surface area was selected based on psychophysiological interaction (PPI) analyses of previous fear extinction functional magnetic resonance imaging (fMRI) studies, which suggested functional connectivity with the vmPFC (MNI: − 6, 36, 12). During extinction learning, brief high-frequency rTMS bursts (300 ms at 20 Hz) were administered online aimed at the left posterior PFC target, time-locked to start 100 ms after the presentation of only one of two CS + . The results demonstrated that this intervention led to significantly reduced fear responses to the TMS-coupled CS + during recall, compared to the CS + not paired with rTMS. In contrast, when rTMS was applied to a control brain region that was not functionally associated with the vmPFC, no significant differences were observed between the two CS + . These findings suggest that high-frequency rTMS targeting the left posterior PFC may be a promising approach to modulate fear extinction and could potentially even improve exposure-based treatment outcomes. However, validation and translational studies are necessary.

The aim of this study was to bridge the gap from laboratory findings in healthy participants to clinical practice by applying TMS to the previously reported left posterior PFC target (MNI: − 56, 2, 40)^35^ in individuals with specific phobias. This translational effort aimed to investigate whether this stimulation target might result in enhanced therapeutic effects and improve our understanding of neural processes involved in fear extinction in specific phobias and their treatment. Thus, active or sham iTBS was administered to the left posterior PFC expected to result in connectivity-based secondary activation of the vmPFC^35^. Offline iTBS was chosen due to its relatively long-lasting neuromodulatory aftereffects^29^ and its suitability for clinical administration. iTBS was applied immediately before two virtual reality exposure (VRET) sessions with a maximum delay of three minutes to ensure enhanced cortical activation during exposure. We hypothesized that patients with acrophobia receiving active iTBS would exhibit significantly greater reductions in acrophobic symptoms compared with sham-stimulated patients, both immediately after the VRET-TMS sessions and at a 6-month follow-up.

## Methods

### Study design

We conducted a two-arm randomized controlled trial (RCT) following a 3 × 2 within- and between-subjects design (within factor: time; between﻿ factor: TMS group; interaction: time x TMS group) with diagnostic assessments conducted before and after two VRET-TMS sessions and a 6-month follow-up. Considering that previous meta-analyses have established high effect sizes for VRET compared to inactive control conditions^36^ and demonstrated comparable or even superior effect sizes relative to active control conditions, such as in-vivo exposure therapy^37^, no control condition for VRET was included in this study. The diagnostic assessments took place in the Department of Psychiatry, Psychosomatics, and Psychotherapy, University Hospital of Wuerzburg, and the VRET-TMS sessions in the 3D-Multisensory Lab (PsyCave) at the Department of Psychology I, University of Wuerzburg. The study protocol was approved by the Ethics Committee of the Medical Faculty of the University of Wuerzburg (proposal number 45/20) and pre-registered at ClinicalTrials.gov (Identifier: NCT04782570) on 04/03/2021. We performed the study according to the guidelines of Good Clinical Practice. All participants gave informed consent according to the Declaration of Helsinki and were compensated 45€.

### Participants

Inclusion criteria were (1) fluent in German language; (2) right-handed; (3) aged between 18 and 65 years; and (4) meeting the diagnostic criteria for acrophobia based on self-reports (according to DSM-5; structured according to the Diagnostic Interview for Mental Disorders^38^). Exclusion criteria were as follows: (1) previous psychotherapeutic or psychiatric treatment for acrophobia; (2) comorbid Axis I disorders and consequently ongoing psychotherapeutic or psychiatric treatment (except for other specific phobias when acrophobia was the primary concern; evaluated with the Diagnostic Short-Interview for Mental Disorders; Mini-DIPS Open Access^39^); (3) use of tricyclic antidepressants, neuroleptics, or medications that increase seizure propensity; (4) severe physical, neurologic, or cardiovascular disorders; (5) increased intracranial pressure; (6) history of severe cranial injury or head surgery; (7) history of seizures or a family history of epilepsy; (8) pregnancy; (9) non-removable metal (e. g., infusion pump, pacemaker) or other MRI-specific contraindications; and (10) non-compliance with the COVID-19 regulations of the University and the University Hospital of Wuerzburg. Clinical physicians carried out physical and neurological examinations at baseline, with no subject having contraindications to the investigational intervention. A detailed description of the participants’ information (e.g., dropout rates, demographic characteristics) can be found in the Results section of this manuscript.

### Study protocol

Participants were primarily recruited through public advertisements from July 2021 to August 2022. The detailed inclusion and exclusion criteria as well as a comprehensive study description were provided during a telephone screening. At baseline, eligible subjects were enrolled with their informed consent by the responsible study investigator, and various data were collected, including demographic, psychometric, behavioral, and epigenetic data (Table 1). The analysis of epigenetic data will not be the subject of this paper but a separate one. In preparation for VRET, subjects received psychoeducational material derived from CBT manuals. This material included information related to the functionality of fear and avoidance, the interaction of cognitive, behavioral, and physiological components, as well as the rationale for exposure therapy (based on a previous study^40^). Participants were instructed to read the manual at home before attending the first VRET visit. Additionally, individual structural MRIs for TMS target navigation were acquired in a separate visit. Participants were then randomly assigned by an independent TMS administrator to either active or sham iTBS (1:1 ratio, gender-balanced). For this purpose, a computer-generated list of individual patient codes was previously generated by an independent study staff member not involved in the assignment, diagnosis, and treatment process. Each code was characterized by either active or sham stimulation. The codes were assigned sequentially and only once within the study. During the first VRET-TMS visit, key psychoeducational information was discussed once, and the upcoming exposure exercise was discussed to ensure that subjects were familiar with the principles of exposure therapy. After the familiarization with VR navigation in a neutral environment without heights, neuronavigated iTBS was delivered in the same room before each of the two exposure therapy sessions. Both post- and follow-up assessments again involved the collection of psychometric, behavioral, and epigenetic outcome data. For the blood samples, participants were instructed to abstain from coffee and exercise before all study time points and to consume a standardized meal.

**Table 1:** Demographic and clinical characteristics at baseline.

	Sham TMS (*n* = 38)	Active TMS (*n* = 38)	Test statistic (*p*)
	*M* (*SD*) or *N* (%)	*M* (*SD*) or *N* (%)	
Age (years)	41.61 (11.86)	37.84 (11.82)	1.39 (.170)
Female	21 (55.26)	21 (55.26)	0.00 (1.000)
AQ			
Anxiety	52.17 (14.52)	53.29 (13.36)	− 0.35 (.729)
Avoidance	33.69 (3.51)	33.17 (4.71)	0.55 (.585)
ATHQ	37.92 (12.58)	36.18 (14.45)	689.00 (.732)
BAT (VR)			
STAI state	38.26 (8.05)	36.52 (7.28)	0.98 (.333)
Max. distance	19.76 (18.30)	22.67 (19.47)	623.50 (.508)
Max. anxiety	65.74 (21.06)	63.33 (23.78)	670.50 (.883)
Presence	60.71 (25.99)	67.78 (27.70)	463.50 (.223)
BAT (in-vivo)			
STAI State	44.74 (12.40)	38.95 (11.12)	2.14 (.035*)
Max. distance	8.39 (3.21)	9.31 (2.84)	612.50 (.228)
Max. anxiety	58.68 (25.62)	61.22 (25.67)	654.00 (.602)
ADS-K	5.14 (3.89)	5.21 (4.02)	720.00 (.983)
ASI 3	17.65 (10.64)	17.08 (8.68)	0.26 (.797)
STAI Trait	33.55 (6.05)	34.95 (8.09)	678.00 (.647)
TAS	46.29 (20.48)	46.39 (22.07)	722.00 (1.000)
Frequency of self-exposure until FU	55.26 (27.29)	53.92 (25.17)	680.50 (.810)

Means and standard deviations or frequency and percent for each TMS group, as appropriate. Test statistics for independent *t*-tests or Mann–Whitney U tests, or *χ*^*2*^, as appropriate, *p < .010.

TMS, transcranial magnetic stimulation; *M*, mean; *SD*, standard deviation; AQ, Acrophobia Questionnaire; ATHQ, Attitudes Towards Heights Questionnaire; BAT, Behavioral Approach Task; VR, Virtual Reality; STAI, State-Trait Anxiety Inventory; ADS-K, German short form of the Center for Epidemiological Studies Depression Scale; ASI 3, Anxiety Sensitivity Index-3; TAS, Tellegen’s Absorption Scale; FU, Follow-Up.

### MRI data processing

**Fig. 1:**
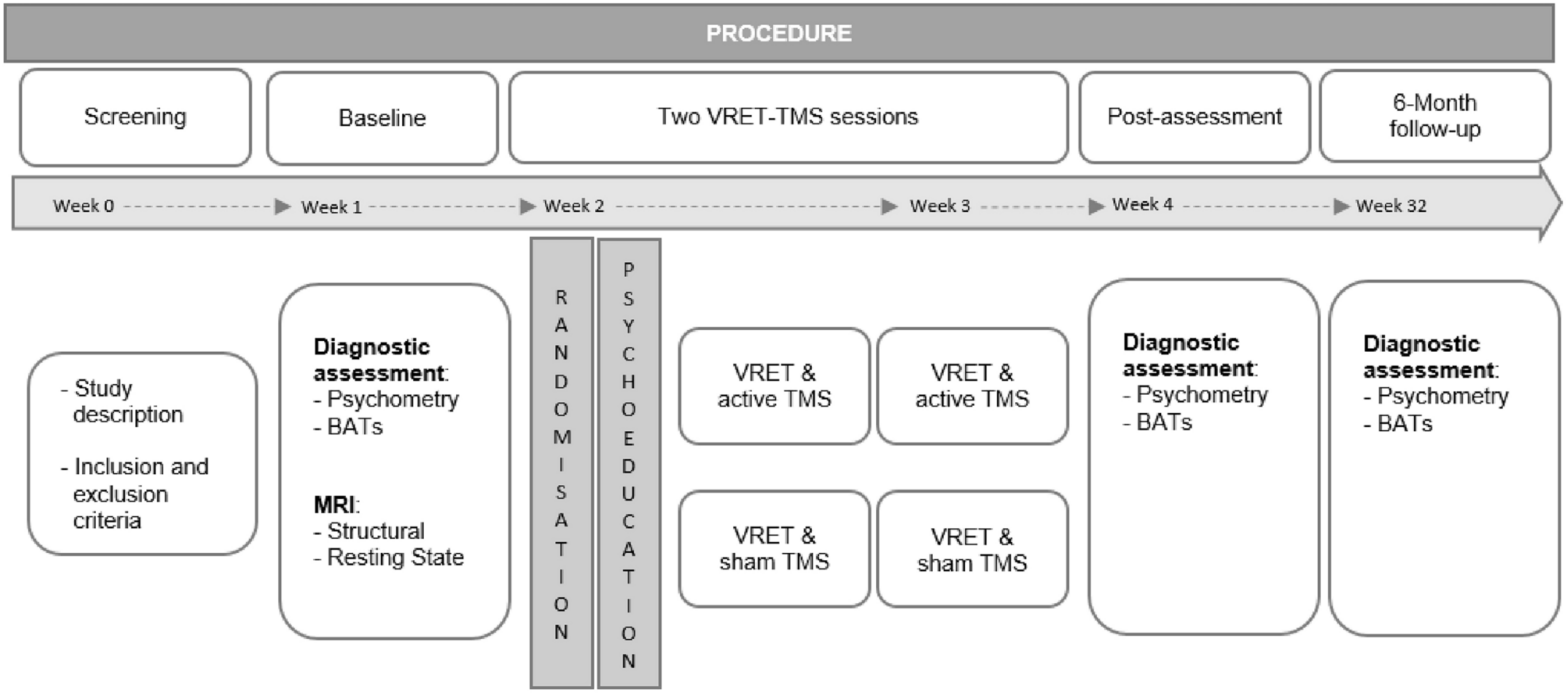
Study design from screening to 6-month follow-up. BAT, Behavioral Approach Task; MRI, magnetic resonance imaging; TMS, transcranial magnetic stimulation; VRET, virtual reality exposure therapy.

All participants underwent T1-weighted anatomical MRI using magnetization-prepared rapid gradient echo (MPRAGE; slices = 176; field of view (FOV) = 256 mm; isotropic voxel size = 1.0 mm^3^; repetition time (TR) = 1900 ms; echo time (TE) = 2.26 ms; flip angle = 9°) and resting-state fMRI (RS-fMRI; layers = 33; FOV = 210 mm; voxel size = 3.3 × 3.3 × 3.8 mm; 10% slice gap; transversal slice orientation; TR = 2000 ms; TE = 29.0 ms; flip angle = 90°) in a 3 T MRI scanner (SIEMENS MAGNETOM Skyra, Siemens Healthcare GmbH, Erlangen, Germany). For both measurements, subjects were asked to close their eyes, to relax, but not to fall asleep. The cortical surface of each participant was reconstructed by default into a surface-based coordinate system (FreeSurfer^41^; vs. 6.0). The cortical surface location with robust connectivity to the vmPFC (see Fig. 1B of the previously described publication^35^) was labeled on the CVS35 average brain with MNI alignment in FreeSurfer and morphed to each individual cortical surface^42^.

### Neuronavigated rTMS

During the first TMS visit, resting motor threshold (rMT) was determined in all subjects over the primary motor cortex using a MagPro X100 stimulator and C-B70 coil (MagVenture GmbH, Willich, Germany; see Table 2 for TMS characteristics in both groups). To match the Cool-B70 A/P coil (MagVenture GmbH, Willich, Germany) used for stimulation of the left posterior PFC, the distance of the C-B70 coil to the head surface was increased by 2 mm with a plate attached to the coil, based on measurements of the induced magnetic field by MagVenture. The appropriate participant code was entered into the TMS device and determined the side to which the coil had to be turned to administer either active or sham TMS. After registration of the subjects into the MRI-based neuronavigation system (Localite GmbH, Bonn, Germany) based on individual structural MRI data, the Cool-B70 A/P coil was positioned at the individual stimulation target, tangential to the scalp and coil rotated perpendicular to the nearest larger sulcus in the MR-based cortical anatomy of the subjects.

**Table 2:** Characteristics for transcranial magnetic stimulation and virtual reality exposure therapy sessions.

	Sham TMS		Active TMS		Sham vs. active TMS	
	*M* (*SD*) or *N* (%)		*M* (*SD*) or *N* (%)		Test statistic (*p*)	
	1st session	2nd session	1st session	2nd session	1st session	2nd session
TMS						
rMT	35.95 (7.31)	–	34.26 (6.06)	–	1.09 (.278)	–
TMS intensity	34.86 (7.00)	35.44 (7.33)	33.68 (5.57)	33.63 (5.67)	0.81 (.421)	1.19 (.118)
VRET						
Duration exposure	23.00 (10.02)	19.79 (6.63)	25.09 (11.29)	20.29 (7.61)	545.50 (.434)	710.50 (.905)
Duration habituation	7.32 (8.35)	5.84 (4.32)	8.13 (8.22)	6.16 (5.14)	644.00 (.415)	693.50 (.919)
Maximal anxiety	90.90 (13.88)	85.66 (20.74)	94.68 (7.32)	91.45 (14.61)	684.00 (.666)	653.50 (.431)
Maximal height						
0–1st level	5 (13.16)	1 (2.63)	4 (10.53)	2 (5.26)	7.40 (.389)	7.55 (.374)
1st level	3 (7.89)	2 (5.26)	3 (7.89)	3 (7.89)		
1st–2nd level	2 (5.26)	1 (2.63)	2 (5.26)	1 (2.63)		
2nd level	6 (15.79)	5 (13.16)	10 (26.32)	3 (7.89)		
2nd–3rd level	0 (0.00)	3 (7.89)	4 (10.53)	2 (5.26)		
3rd level	4 (10.53)	1 (2.63)	4 (10.53)	7 (18.42)		
3rd–4th level	1 (2.63)	0 (0.00)	0 (0.00)	1 (2.63)		
4th level	17 (44.74)	25 (65.79)	11 (28.95)	19 (50.00%)		
Presence	62.76 (21.14)	66.97 (15.00)	67.58 (18.95)	70.20 (15.55)	614.50 (.260)	− 0.92 (.182)
SSQ	21.44 (17.39)	–	23.83 (20.39)	–	646.50 (.684)	–
STAI State	37.41 (9.75)	32.62 (6.86)	39.17 (9.95)	33.86 (8.95)	626.00 (.318)	675.50 (.629)
IPQ						
General presence	4.26 (1.22)	4.29 (0.87)	4.47 (1.25)	4.29 (1.18)	637.00 (.355)	701.00 (.818)
Spatial presence	22.11 (4.10)	21.45 (4.95)	21.82 (4.57)	21.13 (4.13)	693.00 (.762)	0.30 (.764)
Involvement	14.87 (5.51)	15.05 (4.83)	15.32 (5.00)	14.39 (4.50)	− 0.37 (.712)	0.62 (.541)
Experienced realism	11.55 (4.03)	12.37 (4.04)	13.49 (4.63)	13.42 (4.40)	− 1.95 (.055)	− 1.09 (.281)

Means and standard deviations for each TMS group. Test statistics for independent *t*-tests or Mann–Whitney U tests, or *χ*^*2*^, as appropriate.

TMS, transcranial magnetic stimulation; *M*, Mean; *SD*, Standard deviation; rMT, resting motor threshold; VRET, virtual reality exposure therapy; SSQ, Simulator Sickness Questionnaire; STAI, State-Trait Anxiety Inventory; IPQ, Igroup Presence Questionnaire.

iTBS was administered at an intensity of 100% of the individual rMT, including 600 pulses, given in 20 trains. Each train consisted of 10 bursts (3 pulses at 50 Hz per burst) at 5 Hz for 2 s, followed by a pause of 8 s^43^. During the iTBS sessions, the independent TMS administrator recorded the stimulation intensity and made necessary adjustments to address any discomfort experienced by the participants. In the sham TMS condition, no cortical tissue was stimulated, but synchronous auditory artifacts were performed. Additionally, both the active and sham groups received electrical co-stimulation using two 28 × 20 mm surface electrodes (Ambu A/S, Ballerup, Denmark) to ensure double blinding. The intensity of the co-stimulation was technically linked to the individual iTBS intensity and set at 50% of the maximum output. One electrode was placed vertically on Fz and the second on the left at a 90° angle relative to the first electrode at 0.5 cm. During stimulation, the positions of the coil were co-registered to ensure identical placement for the second VRET-TMS visit. At the post-assessment, side effects and participants’ presumed group affiliation were recorded. The unblinding of the group assignment to the participants and the study investigators occurred only after the completion of the follow-up.

### Exposure therapy

For VRET, a five-sided Cave Automatic Virtual Environment (CAVE; 4 × 3 × 2.95 m; I Space, Barco, Kortrijk, Belgium) was used. The CAVE is considered highly immersive and valid for VRET in acrophobia^44^. During the VRET sessions, all subjects underwent a fear-inducing scenario, which involved a tetrahedral-observation tower with see-through metal-mesh stairs and four round platforms (at 18 m, 28 m, 35 m, and 50 m), with the top platform accessible by elevator (developed by VTplus GmbH, Wuerzburg, Germany). For simulation control, the CyberSession software (CS-Research 5.8; VTplus, Wuerzburg, Germany; for more information, see www.cybersession.info) was used in combination with the Source Engine SDK 2013 (Valve, Bellevue, WA, USA) for rendering the VR environment. The VR environment was projected onto four sides and one floor with six Galaxy NW-7 projectors (Barco GmbH, Kortrijk, Belgium) at a resolution of 1920 × 1200 pixels. Participants wore passive interference filter glasses (INFITEC Premium, INFITEC GmbH, Ulm, Germany) for viewing the projected stereoscopic images in 3D. Navigational options within the VR environment included walking or turning oneself or using a wireless Xbox 360 controller (Microsoft Corporation, Redmond, Washington, USA). Sounds were presented with a 7.1 surround sound system.

Participants were instructed to walk from the bottom of the tower as high as possible until they reached maximal anxiety, which was rated on a scale from 0 (for “no anxiety at all”) to 100 (indicating “extremely strong anxiety”). If a participant reported anxiety < 100 but had not yet reached the highest platform, they were instructed to continue ascending. Once either the maximum level of anxiety or the highest platform was reached, anxiety ratings were recorded. Subsequently, participants were instructed to focus on the cue stimulus that triggered the maximum anxiety, while also paying attention to cognitive and physiological changes. They were instructed to wait until their anxiety decreased to a level of 20 or less. During the exposure sessions, VRET parameters including the duration of exposure, duration of habituation, level of maximal anxiety, highest reached platform, and presence in the virtual environment, were recorded (Table 2). After each VRET session, experiences during exposure were discussed with the subjects.

### Measurements

Psychometric and behavioral data for primary and secondary outcomes were collected during each diagnostic assessment. For primary outcomes, acrophobia symptom severity was assessed with the Acrophobia Questionnaire (AQ^45^) consisting of two subscales: “anxiety” and “avoidance”. Both subscales consist of 20 items each, describing height-specific situations (e.g., standing at an open window on the third floor). Participants rated their degree of anxiety related to each situation on a Likert scale from 0 “not at all anxious” to 6 “extremely anxious”, as well as their degree of avoidance from 0 “would not avoid doing it” to 3 “would try to avoid doing it”. Sum scores were calculated separately (total scores for “anxiety”: 0–120; and “avoidance”: 0–40). For secondary psychometric data, the Attitude Towards Heights Questionnaire (ATHQ^46^; total scores: 0–60) assessed further acrophobic symptoms, the Anxiety Sensitivity Index-3 (ASI-3^47^; total scores: 0–72) measured the severity of different concerns related to anxiety symptoms, and the short form of the Center for Epidemiological Studies Depression Scale (ADS-K^48^; total scores: 0–45) assessed depressive symptoms. Secondary behavioral data consisted of two behavioral approach tasks (BATs) to evaluate fear and avoidance in specific height situations, additionally recording the current emotional state (STAI^49^; total scores: 20–80) immediately before both BATs.

The BAT (VR) was carried out using a VR Therapy System (VT + ExpoCart2; VTplus, Wuerzburg, Germany) with a head-mounted display. The VR environment consisted of a glass elevator located on the exterior of a 150 m skyscraper (developed by VTplus, Wuerzburg, Germany). Participants were first familiarized with the VR setup and then instructed to take the elevator as high as possible (1–49 floors), while looking down, without prolonged interruptions and without pushing themselves beyond their comfort level, aiming to prevent an exposure therapy effect. Whenever subjects decided not to proceed or stopped looking down, they were asked to rate their subjective anxiety level on a scale from 0 = “no anxiety at all” to 100 = “extremely strong anxiety”. When subjects reached the 49th floor, they had the opportunity to access the roof terrace (50th floor). At this stage, anxiety ratings were collected at the entrance to the roof terrace, at the railing, and when bending over the railing. Furthermore, VR presence ratings were recorded when participants reached their maximum height.

The BAT (in-vivo) was conducted to assess the generalization of VRET effects in a real-life height situation. The BAT involved an open parking garage stairwell with see-through metal mesh stairs and four levels (at 2.90&nbsp;m, 5.80&nbsp;m, 8.70&nbsp;m, and 11.60&nbsp;m). Participants were instructed to walk from the bottom of the stairwell as high as possible (0–11. 60&nbsp;m) while looking down, without holding onto the railing, without prolonged stops, and without forcing themselves to continue beyond the comfort level. The maximum height reached was determined when participants decided not to proceed further, to hold onto the railing, or were unwilling to look over it at a certain level. Subsequently, participants rated their subjective anxiety on a scale from 0 = “no anxiety at all” to 100 = “extremely strong anxiety”.

In addition to the primary and secondary outcomes, demographic data, trait anxiety (STAI^49^; total scores: 20–80), and hypnotic susceptibility (TAS^50^; total scores: 0–136) were administered at baseline. During the first VRET-TMS visit, symptoms of simulator sickness (SSQ^51^; total scores: 0–235.62) were evaluated after the VR training session. The current emotional state (STAI^49^; total scores: 20–80) before VRET and the sense of presence (IPQ^52^; total scores for “general presence”: 0–6; “spatial presence”: 0–30; “involvement” and “experienced realism”: 0–24) after VRET were assessed in both VRET-TMS sessions. At follow-up, the diagnostic criteria for acrophobia were assessed again using the Diagnostic Interview for Mental Disorders^38^ and the frequency of self-exposure during the last 6 months (0% = “not at all” to 100% = “very frequent”).

### Statistical analysis

The sample size was calculated a priori using GPower 3.1.9.2 (F-tests, ANOVA: repeated measures, within-between interaction) with an effect size of *f* = 0.3 considering our previous study^40^ and systematic review^53^. With two measurements, a type I error of 0.05, and a power of 95%, a final sample size of 76 subjects with 38 participants per group was estimated. However, the main analyses were conducted with linear mixed models (LMMs) for continuous outcomes, or generalized linear mixed models (GLMMs) when assumptions were violated (see Supplementary Table S1), and GLMMs for dichotomous outcomes, as multilevel modeling is a more powerful alternative to conventional mixed ANOVAs and can handle unbalanced and missing data^54^. Fixed factors comprised *time* (within-subject: baseline, post-, and follow-up assessment), *group* (between-subject: sham and active TMS), and the *time x group* interaction. For GLMMs for dichotomous outcomes, the random factor *subjects* was additionally included. Model parameters were estimated using the maximum likelihood method, and degrees of freedom were defined using the Satterthwaite approximation as it controls for Type I error^54^. To examine the additional impact of iTBS on treatment response, subjects with at least a 50% reduction in both AQ subscales (“anxiety” and “avoidance”) from baseline to post-assessment and from baseline to follow-up were defined as responders.

In exploratory analyses, we aimed to estimate the effective connectivity between the vmPFC, which was associated with extinction learning in the previously described study^35^, and all other brain regions, including the individual iTBS target area identified through individual TMS-induced electrical fields. For this, we created head volume conductor models using T1-weighted structural data for each subject in SimNIBS (Version 3.2.6). We simulated the TMS-induced electrical fields (E-fields) using the neuronavigation data, TMS coil characteristics, and iTBS intensities. Subsequently, region-of-interest brain masks were generated based on the simulated E-fields, thresholded at 80%, and the structural MRI data, utilizing the Python module NiLearn (see https://nipy.org/ for more details). For effective connectivity analysis, we preprocessed the structural MRI and RS-fMRI data using the pipeline module of Matlab Toolbox RESTplus (Version 1.28; http://www.restfmri.net). Granger causality analysis (GCA) coefficients were then extracted for the whole brain using the vmPFC (MNI: − 6, 36, 12) as a spherical seed region with a radius of 5 mm and an order of one. To assess the effective connectivity between the stimulation location and the seed region (vmPFC), we only considered voxels within the individualized, thresholded brain masks based on the E-fields simulation. Positive and negative GCA coefficients can be interpreted as predicting increased or decreased activation in one region by activation of another region, reflecting increased or suppressed excitability effects^55^. Next, we performed two-tailed Pearson correlation tests, or Spearman’s rank correlation test when assumptions were violated (see Supplementary Table S5), in the active stimulation group to evaluate the correlative relationship between GCA coefficients (representing effective connectivity between vmPFC and the individual TMS-induced electrical fields) and symptom reduction. Symptom reduction was defined as the decrease in primary outcome scores from baseline to post-assessment and from baseline to follow-up. Furthermore, in the 2nd group-level analyses using data from both groups, we employed the Statistical Parametric Mapping 12 (SPM12; http://www.fil.ion.ucl.ac.uk/spm) software to identify additional significant clusters and peak regions on the cortex based on the GCA coefficients that were adjacent to our stimulated brain area and might serve as potential additional stimulation targets.

Based on previous findings showing a marginally significant correlation between the intensity of TMS and the decrease in subjective distress during BAT from pre- to post-assessment^56^, we conducted exploratory two-tailed Pearson or Spearman’s rank correlation tests (see Supplementary Table S5 for assumption checks). These tests aimed to investigate the association between the mean intensity of iTBS across both TMS sessions and symptom reduction observed in the active iTBS group, both from baseline to post-assessment and from baseline to follow-up.

Statistical analyses were performed in SPSS (IBM SPSS Statistics, Version 29.0, Armonk, New York, USA) on an intention-to-treat (ITT) basis. Group differences in demographic, psychometric, and behavioral data at baseline, VRET and iTBS recordings during the VRET-TMS visits, and further data at follow-up were tested with independent *t*-tests or Mann–Whitney U tests (see Supplementary Tables S2 to S5 for assumption checks) for continuous and *χ*^*2*^-tests for categorical variables. Within- and between-subject effect sizes were calculated using Cohen’s *d* with 95% confidence intervals as pooled standardized differences between estimated marginal means derived from the LMMs or GLMMs. Marginal *R*^*2*^ was derived for fixed effects. All tests were two-tailed, with *p* ≤ .05 considered significant; marginal significant effects were defined between *p* > .05 and *p* ≤ .09. Post-hoc tests were performed with Bonferroni adjustments.

## Results

### Participants

**Fig. 2:**
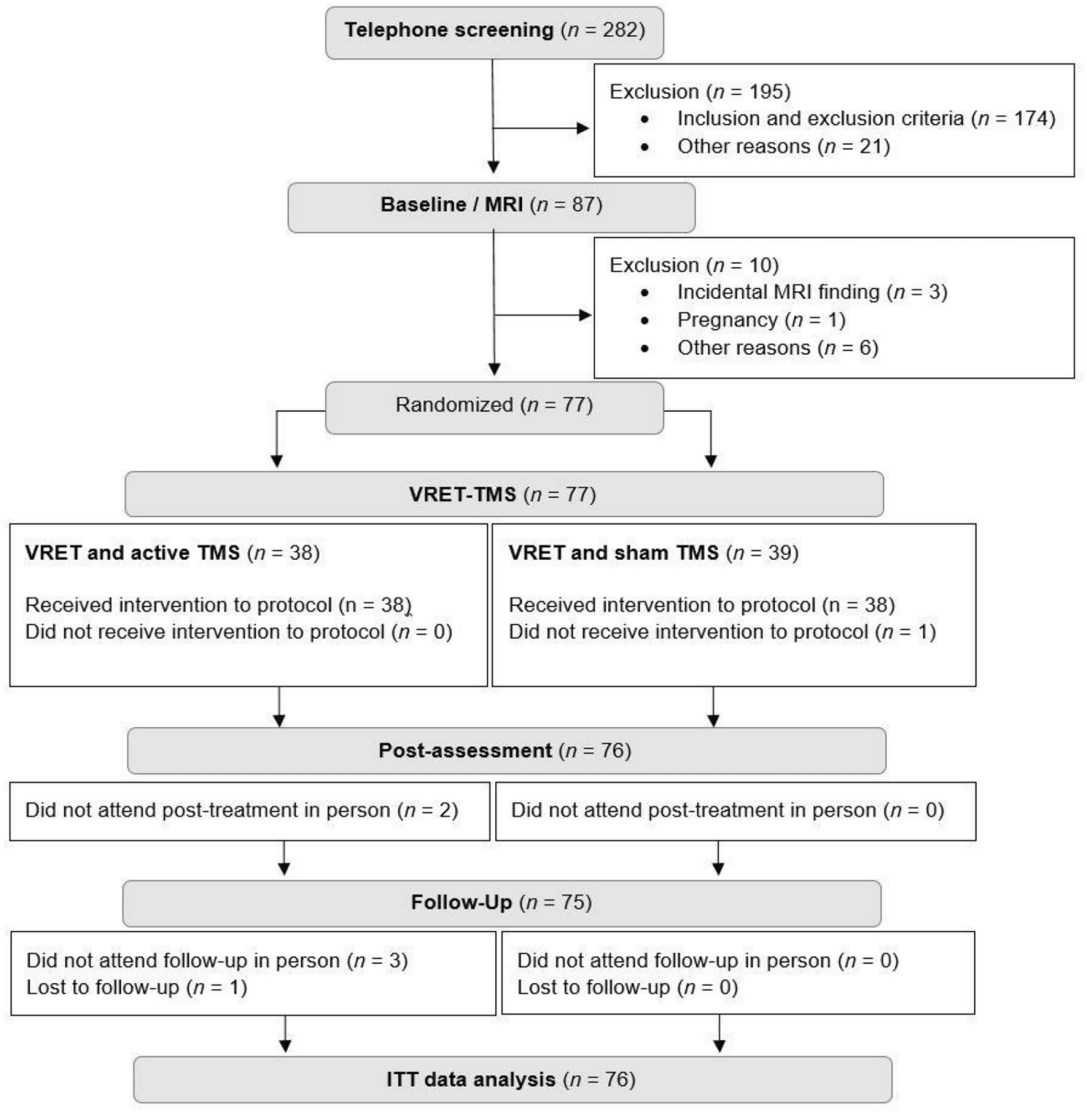
Flowchart diagram. MRI, magnetic resonance imaging; VRET, virtual reality exposure therapy; TMS, transcranial magnetic stimulation; ITT, intention-to-treat.

Overall, 282 individuals were screened for inclusion and exclusion criteria, and *n* = 87 were enrolled in the study (see Fig. 2 for flow chart). *n* = 10 were excluded due to incidental MRI findings (*n* = 3), pregnancy (*n* = 1), high depressions scores (*n* = 1; assessed with ADS-K^48^), loss of contact (*n* = 4), and withdrawal of consent (*n* = 1) after baseline assessment. In total, *n* = 77 underwent active or sham iTBS; *n* = 2 received sham iTBS positioned at F3 of the International EEG 10/20 System and without MRI or neuronavigation. In contrast to the initial study protocol, one subject (from the sham iTBS group) was excluded due to acute illness (unrelated to the study; viral infection) and thus missed the second TMS-VRET session. One subject (active iTBS) dropped out on follow-up because of acute illness (unrelated to the study; virus infection) and loss of contact afterward. Some participants did not attend post-assessment (*n* = 2; from the active TMS group) and follow-up (*n* = 3; from the active TMS group) but sent psychometric data via e-mail and were therefore included in the data analysis. Therefore, a total of 76 subjects, *n* = 38 in each group, were included in the data analysis.

Except for the follow-up assessment 6 months later, all visits were scheduled weekly (days between first and second VRET-TMS visits: *M*_sham_ = 7.18, *SD* = 2.13; *M*_active_ = 7.34, *SD* = 2.20; *U* = 697.50, *Z* = − 0.26, *p* = .795; days between second VRET-TMS visit and post-assessment: *M*_sham_ = 6.66, *SD* = 2.60; *M*_active_ = 6.31, *SD* = 2.52; *U* = 662.00, *Z* = − 0.24, *p* = .810; days between post-assessment and follow-up: *M*_sham_ = 191.68, *SD* = 16.99; *M*_active_ = 193.12, *SD* = 17.02; *U* = 578.50, *Z* = − 0.76, *p* = .446). Participants did not differ significantly between the active and sham iTBS groups in demographic, psychometric, behavioral data at baseline, VRET and iTBS parameters, and self-exposure frequency until follow-up (Tables 1 and 2 for details). Only for STAI scores before BAT (in-vivo), participants from the sham group showed higher anxiety levels (*M*_sham_ = 44.74, *SD* = 12.40) compared to the active group (*M*_active_ = 38.95, *SD* = 11.12; *t*(74.00) = 2.14, *p* = .035). These baseline differences might influence further analyses and results should be interpreted cautiously. In addition, a marginally significant difference in the “experienced realism” subscale of the IPQ in the first VRET-TMS visit was found (*t*(74.00) = − 1.95, *p* = .055; see Table 2 for more details). At follow-up, 10.34% of the active iTBS group still fulfilled diagnostic criteria for a specific phobia, compared with 5.88% in the sham iTBS group (*χ*^*2*^(1) = 0.43, *p* = .514).

### iTBS administration

The observed side effects were similar between the active and sham groups (*χ*^*2*^(1) = 2.73, *p* = .098). No serious adverse events or epileptic seizures were reported at the post- or follow-up assessment. The most frequent side effect was headache (sham TMS: 21.05%; active TMS: 39.47%), which was expected. The groups did not differ significantly for headaches intensity (*M*_sham_ = 5.13, *SD* = 2.53; *M*_active_ = 4.08, *SD* = 1.50; *t*(19.00) = 1.20, *p* = .122). Other side effects, e.g., dizziness (sham TMS: 2.63%; active TMS: 15.79%), nausea (sham TMS: 5.26%; active TMS: 5.26%), or neck pain (sham TMS: 2.63%; active TMS: 5.26%) were also described at post-assessment. However, the groups did not differ significantly in the reported intensities (all *p*’s > .050). One should note that the adverse effects might also have occurred from VRET. Furthermore, no association between the actual TMS condition and the guess of TMS assignment, *χ*^*2*^(1) = 1.73, *p* = .188, was found. Overall, 71.05% of the placebo TMS group and 83.78% of the active TMS group guessed that they obtained active stimulation. In the active-stimulated group, TMS intensity had to be adjusted in *n* = 5 during the first VRET-TMS visit (mean TMS intensity after adjustment; % rMT: *M* = 89.69, *SD* = 5.63), and *n* = 4 for the second VRET-TMS visit (*M* = 85.50 *SD* = 11.48). No significant correlation was found in the active TMS group between iTBS intensity and symptom reduction from baseline to post-assessment (AQ; anxiety: *ρ* = − 0.07, *p* = .667; avoidance: *r* = 0.10, *p* = .572) and to follow-up (anxiety:* r* = 0.05, *p* = .792; avoidance: *r* = 0.05, *p* = .780).

### Primary outcomes

**Fig. 3:**
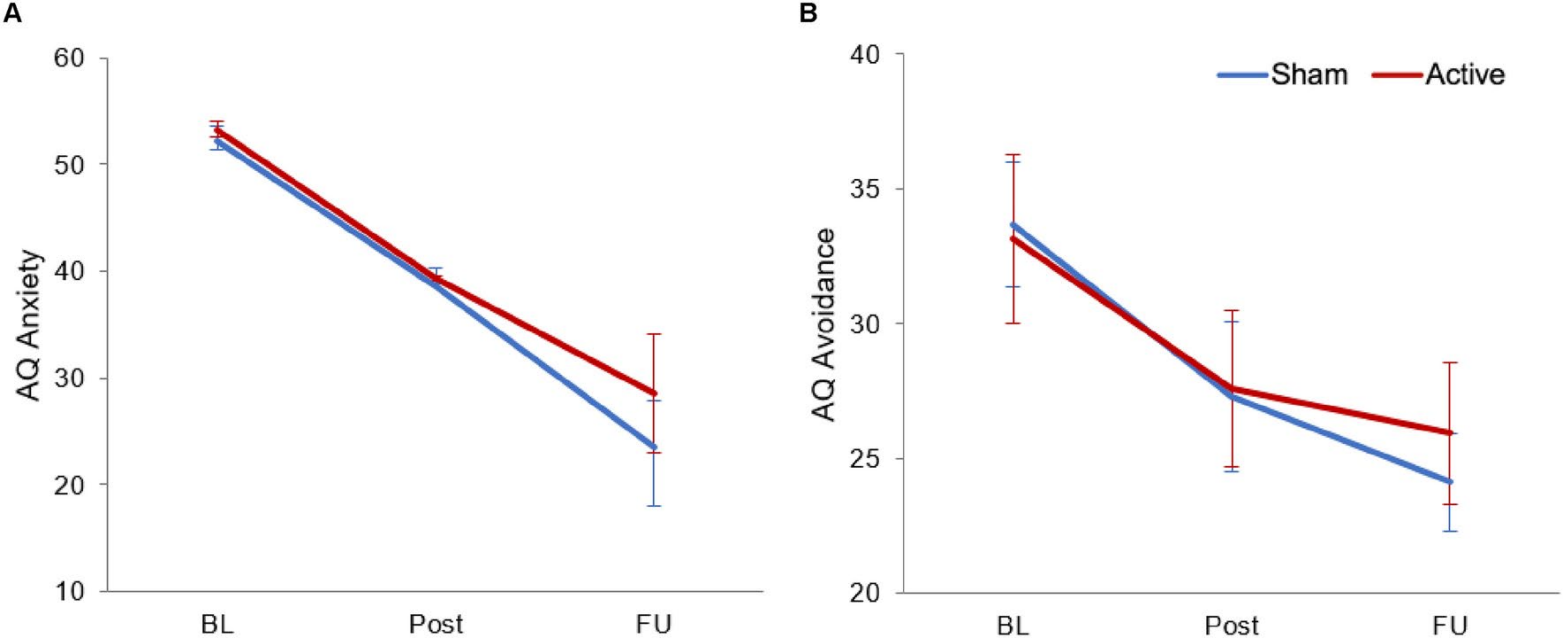
Average trajectories in means with 95% confidence intervals for primary outcome scores. (**A**) Trajectories for Acrophobia Questionnaire (AQ) subscale “anxiety” (total scores: 0–120). (**B**) Trajectories for AQ subscale “avoidance” (total scores: 0–40). Both trajectories from baseline to post-assessment and follow-up for both TMS groups. BL, Baseline; Post, Post-assessment; FU, Follow-up.

Both groups showed significant symptom reduction in primary outcomes from baseline to post and follow-up assessment (AQ; anxiety: *F*(1, 227.00) = 63.60, *p* < .001; avoidance: *F*(2, 220.00) = 82.11, *p* < .001; see Fig. 3 and Table 3 for more details). This effect was observed for baseline to post-assessment (anxiety: *β* = − 13.54, *SE* = 3.33, *t*(227.00) =  − 4.06, *p* < .001, *d*_*within*_ = − 0.67, 95% CI [− 1.00, − 0.34]; avoidance: *β* = − 6.38, *SE* = 0.96, *t*(220.00) =  − 6.64, *p* < .001, *d*_*within*_ = − 1.01, 95% CI [− 1.35, − 0.67]) and from baseline to follow-up (anxiety: *β* = − 28.60, *SE* = 3, *t*(227.00) =  − 8.58, *p* < .001, *d*_*within*_ = − 1.30, 95% CI [− 1.65, − 0.95]; avoidance: *β* = − 9.58, *SE* = 0.92, *t*(226.00) = − 10.43, *p* < .001, *d*_*within*_ = − 1.47, 95% CI [− 1.83, − 1.11]). However, contrary to our hypotheses, no significant *time x group* interaction was found (all *p*’s > .050; see Table 3).

**Table 3:** Average trajectories in means and standard deviations for primary and secondary outcomes from baseline to post-assessment and follow-up.

Outcome	Sham TMS					Active TMS					Sham vs. Active TMS					
	*M* (*SD*) or *N* (%)			Within ES		*M* (*SD*) or *N* (%)			Within ES		Between ES		Time		Time x TMS group	
	BL	Post	FU	BL to Post	BL to FU	BL	Post	FU	BL to Post	BL to FU	Post	FU	*F*	*p*	*F*	*p*
AQ																
Anxiety	52.17 (14.52)	38.63 (17.87)	23.57 (12.66)	− 0.66 [− 1.12,− 0.20]	− 1.39 [− 1.89,− 0.89]	53.29 (13.36)	39.32 (15.02)	28.54 (14.40)	− 0.68 [− 1.14,− 0.22]	− 1.20 [− 1.70,− 0.71]	0.03 [− 0.42,0.49]	0.05 [− 0.40,0.50]	63.60	< .001***	0.50	.610
Avoidance	33.69 (3.51)	27.31 (4.24)	24.11 (2.73)	− 1.08 [− 1.56,− 0.60]	− 1.69 [− 2.22,− 1.17]	33.17 (4.71)	27.56 (4.41)	25.92 (3.99)	− 0.95 [− 1.43,− 0.47]	− 1.26 [− 1.76,− 0.76]	0.05 [− 0.40,0.50]	0.37 [− 0.08,0.83]	82.11	< .001***	1.79	.169
ATHQ	37.92 (12.58)	26.84 (9.52)	19.45 (9.84)	− 0.56 [− 1.02,− 0.10]	− 0.91 [− 1.39,− 0.44]	36.18 (14.45)	22.55 (11.33)	21.46 (11.51)	− 0.72 [− 1.18,− 0.26]	− 0.82 [− 1.29,− 0.35]	− 0.19 [− 0.64,0.26]	0.11 [− 0.34,0.56]	28.39	< .001***	0.87	.420
ADS-K	5.14 (3.89)	3.95 (2.79)	4.04 (2.93)	− 0.30 [− 0.75,0.15]	− 0.19 [− 0.64,0.26]	5.21 (4.02)	5.16 (3.81)	5.49 (4.19)	0.05 [− 0.40,0.50]	0.17 [− 0.28,0.63]	0.30 [− 0.15,0.76]	0.30 [− 0.15,0.76]	0.73	.483	1.52	.222
ASI-3	17.65 (10.64)	14.34 (8.23)	11.57 (6.06)	− 0.23 [− 0.68,0.22]	− 0.46 [− 0.92,− 0.01]	17.08 (8.68)	14.71 (8.64)	12.67 (9.04)	− 0.17 [− 0.62,0.28]	− 0.27 [− 0.72,0.18]	0.03 [− 0.42,0.48]	0.16 [− 0.29,0.62]	5.04	.007*	0.33	.719
BAT (VR)																
STAI State	38.26 (8.05)	29.97 (6.92)	27.22 (4.96)	− 0.79 [− 1.27,− 0.32]	− 1.08 [− 1.57,− 0.60]	36.52 (7.28)	30.60 (8.48)	28.36 (7.78)	− 0.57 [− 1.04,− 0.10]	− 0.81 [− 1.30,− 0.32]	0.07 [− 0.39,0.52]	0.13 [− 0.33,0.60]	33.16	< .001***	0.78	.462
Max. Distance	19.76 (18.30)	32.61 (19.36)	34.86 (18.89)	0.41 [− 0.04,0.87]	0.46 [0.00,0.92]	22.67 (19.47)	31.06 (18.12)	37.29 (18.67)	0.27 [− 0.20,0.73]	0.41 [− 0.07,0.88]	− 0.04 [− 0.50,0.41]	0.06 [− 0.41,0.52]	8.66	< .001***	0.19	.824
Max. Anxiety	65.74 (21.06)	47.68 (24.45)	34.11 (25.03)	− 0.37 [− 0.82,0.09]	− 0.59 [− 1.05,− 0.12]	63.33 (23.78)	55.61 (28.11)	43.91 (30.17)	− 0.12 [− 0.58,0.34]	− 0.34 [− 0.81,0.13]	0.21 [− 0.25,0.66]	0.22 [− 0.25,0.69]	7.96	< .001***	0.60	.551
BAT (in-vivo)																
STAI State	44.74 (12.40)	31.63 (7.62)	26.77 (5.48)	− 0.97 [− 1.45,− 0.49]	− 1.43 [− 1.96,− 0.91]	38.95 (11.12)	31.05 (8.19)	26.69 (6.91)	− 0.64 [− 1.11,− 0.18]	− 1.04 [− 1.53,− 0.55]	− 0.05 [− 0.51,0.40]	− 0.01 [− 0.49,0.47]	55.49	< .001***	2.15	.120
Max. Distance	8.39 (3.21)	10.39 (2.05)	10.79 (1.64)	0.52 [0.06,0.98]	0.62 [0.14,1.11]	9.31 (2.84)	10.56 (2.26)	10.77 (1.96)	0.31 [− 0.15,0.77]	0.35 [− 0.12,0.81]	0.04 [− 0.42,0.50]	0.00 [− 0.48,0.47]	10.55	< .001***	0.60	.552
Max. Anxiety	58.68 (25.62)	41.58 (29.30)	24.19 (21.37)	− 0.24 [− 0.69,0.21]	− 0.67 [− 1.15,− 0.18]	61.22 (25.67)	45.28 (26.59)	28.16 (31.75)	− 0.29 [− 0.75,0.17]	− 0.47 [− 0.94,− 0.01]	0.00 [− 0.45,0.46]	0.21 [− 0.27,0.68]	11.78	< .001***	0.31	.731

Within effect sizes (Cohen’s *d*) for both TMS groups and between effect sizes (Cohen’s *d*) with 95% confidence interval, *F*-statistic for linear mixed models or generalized linear mixed models, * *p* < .050, *** *p* < .001, marginal R^2^ for fixed effects.

TMS, transcranial magnetic stimulation; *M*, Mean; *SD*, Standard deviation; ES, Effect sizes; BL, Baseline; Post, Post-assessment; FU, Follow-up; AQ, Acrophobia Questionnaire; ATHQ, Attitudes Towards Heights Questionnaire; ADS-K, German short form of the Center for Epidemiological Studies Depression Scale; ASI-3, Anxiety Sensitivity Index-3; BAT, Behavioral Approach Task; VR, Virtual Reality; STAI, State-Trait Anxiety Inventory.

### Secondary outcomes

In all secondary outcomes, significantly lower anxiety symptoms were found from baseline to post-assessment and to follow-up (all *p’*s < .010, *d* = − 0.95 to 0.48), but no significant *time x group* interactions (all *p’*s > .050) were found (see Table 3 for more details). Solely for ADS-K, no significant main effect *time* and interaction *time x group* were found (all *p*’s > .050).

### Additional iTBS effect on treatment response

No significant group differences were found regarding the treatment response for the subscale “anxiety” at post-assessment (sham TMS: 26.30%; active TMS: 13.20%; *p* = .160), and follow-up (sham TMS: 55.30%; active TMS: 36.80%; *p* = .139). For the subscale “avoidance”, no participant was characterized as a responder for post and follow-up assessment (sham TMS: 0.00%; active TMS: 0.00%).

### Effective connectivity

**Fig. 4:**
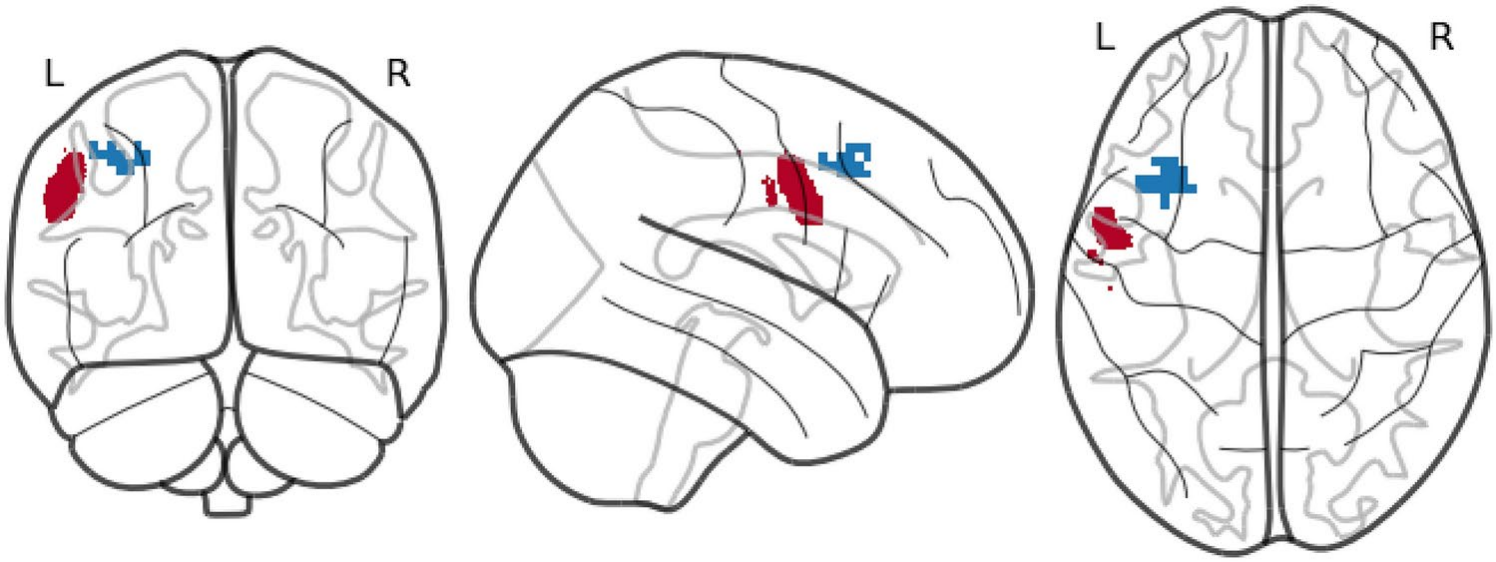
TMS-induced electrical field binary mask (in red) and significant cluster (in blue; MNI: − 36, 15, 35; cluster size = 31) adjacent to the stimulated brain area and showing effective connectivity to the ventromedial prefrontal cortex (vmPFC; MNI: − 6, 36, 12) Average binary mask for all subjects. Cluster-based on GCA coefficients. L, left; R, right.

In the active TMS group, no significant Pearson correlation was found between GCA coefficients and symptom reduction (AQ; anxiety: *ρ* = − 0.05, *p* = .781; avoidance: *ρ* = − 0.10, *p* = .556). However, at the group level, another significant Bonferroni-corrected cluster (MNI: − 36, 15, 45; cluster size = 31, T = 4.52, *p* = .043) accessible with TMS was identified in proximity to the individually stimulated left posterior prefrontal cortex (MNI: − 56, 2, 40; see Fig. 4).

## Discussion

This study was the first RCT attempting to translate promising previous findings in which rTMS was delivered to the left posterior PFC during extinction learning in healthy subjects^33^, into therapeutic application in anxiety disorders. In this study, we administered an offline iTBS protocol over the individual left posterior PFC immediately before two sessions of VRET in acrophobic participants. Both the active and sham TMS groups exhibited significant reductions in acrophobic symptoms at both post-assessment and follow-up with pre-post effect sizes ranging from Cohen’s *d* = 0.12 to 1.69. Specifically, at baseline, both groups showed mean AQ “anxiety” scores exceeding the cut-off of 45.45, which is indicative of highly anxious and acrophobic subjects^57^. Following the VRET-TMS sessions, both groups achieved mean “anxiety” levels below this cut-off, with scores at follow-up similar to those observed in healthy individuals^57^. Our results may demonstrate the viability and comparability of VRET as an alternative to in-vivo exposure therapy, providing further validation of previous meta-analytic findings that show comparable or even superior effect sizes^37^. Additionally, VRET offers the advantage of a standardized setting for treatment. However, since no control condition for VRET was incorporated in this study, the observed anxiolytic effects may be attributable to alternative factors (e.g., therapeutic alliance or the extended engagement with professionals during the study, which could have fostered expectations of improvement among participants).

Interestingly, contrary to our hypotheses, no significant additional benefit was found in the active iTBS group compared with the sham iTBS group. These results are inconsistent with the findings of our previous study, in which a 10-Hz offline rTMS was applied over the medial PFC (mPFC; Fpz of the 10–20 EEG system; 1560 pulses, 100% of rMT; based on a previous laboratory study^58^) immediately before two VRET sessions using an identical VR scenario in a clinical sample with acrophobia^40^. The group receiving active stimulation exhibited significantly greater reductions in acrophobic symptoms from pre- to post-assessment compared to the sham rTMS group. Supporting these findings, a pilot study conducted on patients with spider phobia indicated a marginally significant correlation between rTMS intensity over the mPFC and improved subjective distress during BAT^56^. Additionally, an fMRI study revealed that rTMS applied over the mPFC significantly enhanced functional connectivity between the medial frontal cortex and amygdala^59^. Overall, these previous findings provided encouraging evidence for rTMS over the mPFC as a stimulation protocol for modulating fear extinction during exposure therapy. However, several factors might have contributed to the discrepancy between our present findings and those of previous studies reporting beneficial TMS effects.

One potential explanation might be that our TMS protocol over the left posterior PFC did not induce secondary activation of the vmPFC. Although the PPI-fMRI analyses suggested functional connectivity between the left posterior PFC and the vmPFC^35^, diffusion MRI connectivity analyses did not show direct connections between these regions. Accordingly, the previously reported modulatory effects might have resulted from an indirect activation of the vmPFC mediated by alternative brain areas or even directly from neural circuits not involving additional vmPFC activation^35^. Moreover, functional connectivity patterns differ between healthy individuals and patients with anxiety disorders^20,21^. Thus, our TMS protocol might have modulated other underlying neural networks not functionally connected to the vmPFC or other brain structures involved in fear extinction processes. This assumption is supported by our exploratory GCA analyses, which demonstrated no significant correlative association between the effective connectivity of the individually stimulated left posterior PFC and the vmPFC, and the reduction of phobic symptoms. Accordingly, it is plausible that selecting a different superficial brain area with robust neural connectivity to the vmPFC and modulatory effects on treatment outcomes might have been more appropriate in our clinical population. Interestingly, our exploratory analysis revealed another cluster (MNI: − 36, 15, 45) showing significant effective connectivity to the vmPFC. The proximity of this cluster to the targeted left posterior PFC suggests that TMS over the targeted posterior PFC might have modulated vmPFC activation, hinting at the possibility that our stimulation might have been slightly off-target due to intraindividual variability in neuronal structure, functional connectivity, or effective connectivity. Overall, these findings emphasize the necessity for further investigations into TMS targeting based on peak activation derived from individual effective or functional connectivity between the vmPFC and cortical surface areas, as well as electric field simulation, to define individualized stimulation targets. Furthermore, our exploratory analyses included MNI coordinates of the vmPFC from previous findings of another healthy cohort^35^. Defining the individual ventromedial seed region during fear extinction to identify the individual superficial peak region through estimates of effective connectivity would have yielded more accurate results and should be considered in future investigations. Although this approach may present practical challenges in standard clinical practice, it is a necessary compromise to provide substantial evidence for subsequent translational and clinical applications of TMS^29^, particularly in the advancement of personalized TMS treatment modalities.

Furthermore, it is plausible that the observed modulatory effects over the left posterior PFC were affected by employing an offline TMS protocol instead of an online protocol. While some studies have provided evidence for offline stimulation to enhance fear extinction and treatment effects in anxiety disorders, some other studies have reported detrimental effects (for further details, see reviews^26,34^). For instance, in a rodent model, the effects of electrical stimulation bursts of the IL cortex on fear recall were found only when the cortical stimulation occurred exactly at 100–400 ms after CS + presentation and disappeared if this tight temporal coupling was removed^32^. In another rodent study, the comparison of offline (before extinction learning) and online (during extinction learning) rTMS stimulation protocols over the IL demonstrated that stimulation before extinction learning did not lead to significant improvements in fear extinction compared to online stimulation^60^. To date, no study has investigated the effects of offline iTBS over the left posterior PFC in healthy individuals; thus the optimal TMS parameters for this purpose remain unexplored. Our present results indicated that the beneficial effects of an online rTMS protocol over the left posterior PFC on fear extinction may not be directly transferable to an offline iTBS protocol for specific phobias. Hence, future studies should consider incorporating online TMS protocols that are time-locked to the presentation of phobic stimuli within VR environments. Such studies could provide valuable insights into optimizing TMS interventions for anxiety disorders in exposure therapy.

Conversely, it is also plausible that our TMS protocol induced connectivity-based activation of the vmPFC, but the neural connections between the vmPFC and our targeted TMS region were underestimated in our post-hoc analyses using RS-fMRI. Future studies incorporating robust PPI analyses of task-based MRI measures during fear extinction, along with validating direct stimulation effects on individual target regions and their subordinated brain networks e.g., in concurrent TMS-fMRI studies, are warranted to enhance the evidence for TMS in psychotherapy.

Assuming that the vmPFC was indeed activated through our TMS protocol, our study underscores previous findings that highlight the challenges associated with transferring TMS effects from healthy subjects to specific phobias. For instance, a laboratory study examined the effects of active and sham iTBS administered before and after extinction learning over the left dorsolateral PFC (dlPFC; F3 of the international 10–20 EEG system) on extinction recall in healthy subjects^61^. The dlPFC has also been discussed in the literature as a potential stimulation target to enhance fear extinction processes in the treatment of anxiety disorders, given that patients with anxiety disorders also exhibit dysfunctional activation patterns in this specific brain region^21^. The results of this study indicated that subjects who received active iTBS either before or after extinction learning showed significantly reduced fear responses during extinction recall compared to the sham iTBS group. However, in a study involving patients with spider phobia, a single session of iTBS applied over the left dlPFC (F3 of the international 10–20 EEG system) preceding a VR challenge, where several spiders were presented to participants, did not lead to significant modulations in subjective and physiological fear parameters^62^. Acknowledging the influence of inter- and intraindividual factors on the neurophysiological NIBS effects, such as genetic variations and polymorphisms ^63^, initial neural activation^64^, or experimental task performance, is crucial. For example, a study demonstrated variations in the effects of NIBS on motor-evoked potentials when subjects performed short-lasting motor contractions^65^. Accordingly, TMS effects might differ between healthy and clinical subjects, who could differ in arousal levels or undergo distinct experimental tasks, such as viewing stimuli on a computer screen in the laboratory versus interacting with a virtual environment during VRET, among other differences in the experimental setups. Furthermore, in classical fear conditioning paradigms, participants are typically not confronted with the CS without the UCS before extinction learning (analogous to both BATs), are not prepared for extinction learning (analogous to psychoeducation), and there is typically no discussion of their experiences during extinction learning afterward (analogous to the discussion following VRET). These deviations from laboratory fear extinction paradigms may have influenced participants’ expectations during VRET and cognitive processing during or after the sessions, as well as the impact of TMS on VRET effects. While such differences should not impede translational research on NIBS, they should be systematically considered in future studies to improve our understanding of their impact on TMS interventions^29^.

Moreover, the previously described TMS protocols are typically evaluated within laboratory settings using conditioned fear memory paradigms. While fear conditioning models provide valuable translational insights into anxiety disorders, there are limitations in directly translating fear memory mechanisms to those observed in anxiety disorders^10^. Even in specific phobias, where an explicit aversive experience with the phobic stimulus can be expected as a triggering factor, only a subset of patients (ranging from 18% to 57.5%) are able to identify such an event^66^. This suggests that phobic symptoms may also develop through alternative processes, such as fear acquisition occurring via socially mediated information^67^, involving additional neural mechanisms beyond those in classical fear conditioning paradigms. Hence, it is plausible that the implementation of an online TMS protocol specifically targeting a classical fear memory might present challenges when applied to a phobic memory that has been consolidated over several years during VRET. Looking forward, research has provided other constructs and processes beyond fear extinction, including cognitive reappraisal, self-efficacy, counterconditioning, or interrupting fear reconsolidation processes^9,10^. For example, a study showed that TMS administered over the dlPFC during the reconsolidation phase (10 min after fear reactivation) resulted in significant fear reduction 24 h after stimulation compared with controls^68^. To improve the development of effective TMS protocols for patients, it is imperative to consider these further factors and processes in future basic studies and translational clinical trials.

Finally, it should be acknowledged that the comparable “anxiety” scores (AQ) observed in our subjects at follow-up and in healthy individuals^57^ could suggest the presence of a ceiling effect of VRET in acrophobia, which might have contributed to the absence of an additional long-term TMS effect. Therefore, there is a necessity to examine the impact of TMS particularly in anxiety disorders where CBT exhibits lower efficacy, such as panic disorders with agoraphobia^6^. It might be worthwhile to assess these effects by employing a reduced number of exposure sessions.

In conclusion, although iTBS over the left posterior PFC had no additional effect in the treatment of acrophobia, the present study provides valuable insights into the feasibility and efficacy of combining TMS with exposure-based CBT and opens new approaches for future research. To advance the field, further investigations are warranted in clinical populations with lower CBT efficacy, while considering TMS targeting at both group and individual levels, including individual effective and functional brain connectivity. Additionally, online TMS protocols should be carefully adapted and validated for (online) administration in exposure therapy, with the inclusion of robust placebo controls, sufficient statistical power, and consideration of methodological and inter- and intraindividual variability. Moreover, the incorporation of multiple outcome measures, encompassing physiological and subjective assessments, will enhance the transferability of translational validation studies. By addressing these challenges and also considering alternative anxiolytic mechanisms, TMS exhibits potential as a promising therapeutic approach for the treatment of anxiety disorders.

## Supplementary Information

Supplementary Information.

## Data Availability

Datasets generated during and/or analyzed during the current study are available on reasonable request.
