# Supplementary material for: Intermittent theta burst stimulation over the left prefrontal cortex: no additional effect for virtual reality exposure therapy in acrophobia—a randomized trial
Source: Sci Rep. 2024 Nov 27;14:29450. doi: 10.1038/s41598-024-80832-1 (PMC11603325; doi:10.1038/s41598-024-80832-1)

Supplementary materials

Table S1

Skewness, kurtosis, and normality tests for linear mixed models for primary and secondary outcomes for each TMS group. Test statistics for Shapiro-Wilk Test, * *p* < .050, ** *p <* .010, *** *p <* .001. Abbreviations: TMS, transcranial magnetic stimulation; *SE*, standard error; AQ, Acrophobia Questionnaire; ATHQ, Attitudes Towards Heights Questionnaire; BAT, Behavioral Approach Task; VR, Virtual Reality; STAI, State-Trait Anxiety Inventory; ADS-K, German short form of the Center for Epidemiological Studies Depression Scale; ASI 3, Anxiety Sensitivity Index-3; TAS, Tellegen's Absorption Scale.

Table S2

Skewness, kurtosis, and normality tests for demographic, psychometric, and behavioral data at baseline for each TMS group (used for independent t-tests). Test statistics for Shapiro-Wilk Test, * *p* < .050, ** *p <* .010, *** *p <* .001. Abbreviations: TMS, transcranial magnetic stimulation; *SE*, standard error; AQ, Acrophobia Questionnaire; ATHQ, Attitudes Towards Heights Questionnaire; BAT, Behavioral Approach Task; VR, Virtual Reality; STAI, State-Trait Anxiety Inventory; ADS-K, German short form of the Center for Epidemiological Studies Depression Scale; ASI 3, Anxiety Sensitivity Index-3; TAS, Tellegen's Absorption Scale.

Table S3

Skewness, kurtosis, and normality tests for VRET and iTBS parameters during the first VRET-TMS visit for each TMS group (used for independent *t*-tests).Test statistics for Shapiro-Wilk Test, * *p* < .050, ** *p <* .010, *** *p <* .001. Abbreviations: TMS, transcranial magnetic stimulation; *SE*, Standard error; rMT, resting motor threshold; VRET, virtual reality exposure therapy; SSQ, Simulator Sickness Questionnaire; STAI, State-Trait Anxiety Inventory; IPQ, Igroup Presence Questionnaire.

Table S4

Skewness, kurtosis, and normality tests for VRET and iTBS parameters during the second VRET-TMS visit for each TMS group (used for independent *t*-tests).Test statistics for Shapiro-Wilk Test, * *p* < .050, ** *p <* .010, *** *p <* .001. Abbreviations: TMS, transcranial magnetic stimulation; *SE*, Standard error; rMT, resting motor threshold; VRET, virtual reality exposure therapy; SSQ, Simulator Sickness Questionnaire; STAI, State-Trait Anxiety Inventory; IPQ, Igroup Presence Questionnaire.

Table S5

Skewness, kurtosis, and normality tests for further data for each TMS group (used for independent *t*-tests).Test statistics for Shapiro-Wilk Test, * *p* < .050, ** *p <* .010, *** *p <* .001. Abbreviations: TMS, transcranial magnetic stimulation; *SE*, standard error; VRET, virtual reality exposure therapy; Post, Post-assessment; FU, Follow-Up; GCA, Granger causality analysis; BL, Baseline; AQ, Acrophobia Questionnaire.

Figure S1

Average trajectories in means with 95% confidence intervals for Attitudes Towards Heights Questionnaire (ATHQ; total scores 0-60). Trajectories from baseline to post-assessment and follow-up for both TMS groups. Abbreviations: BL, Baseline; Post, Post-assessment; FU, Follow-up.

Figure S2

Average trajectories in means with 95% confidence intervals for the short form of the Center for Epidemiological Studies Depression Scale (ADS-K; total scores 0-45). Trajectories from baseline to post-assessment and follow-up for both TMS groups. Abbreviations: BL, Baseline; Post, Post-assessment; FU, Follow-up.

Figure S3

Average trajectories in means with 95% confidence intervals for the Anxiety Sensitivity Index-3 (ASI-3; total scores 0-72). Trajectories from baseline to post-assessment and follow-up for both TMS groups. Abbreviations: BL, Baseline; Post, Post-assessment; FU, Follow-up.

Figure S4

Average trajectories in means with 95% confidence intervals for the current emotional state before the behavioral approach task (BAT) in virtual reality (VR) using the subscale State of the State-Trait Anxiety Inventory (STAI State; total scores 0-80). Trajectories from baseline to post-assessment and follow-up for both TMS groups. Abbreviations: BL, Baseline; Post, Post-assessment; FU, Follow-up.

Figure S5

Average trajectories in means with 95% confidence intervals for the maximal subjective anxiety level (scale from 0 to 100) during the behavioral approach task (BAT) in virtual reality (VR). Trajectories from baseline to post-assessment and follow-up for both TMS groups. Abbreviations: BL, Baseline; Post, Post-assessment; FU, Follow-up.

Figure S6

Average trajectories in means with 95% confidence intervals for the maximal distance achieved (from level 0 to 50) during the behavioral approach task (BAT) in virtual reality (VR). Trajectories from baseline to post-assessment and follow-up for both TMS groups. Abbreviations: BL, Baseline; Post, Post-assessment; FU, Follow-up.

Figure S7

Average trajectories in means with 95% confidence intervals for the current emotional state before the behavioral approach task (BAT) in-vivo using the subscale State of the State-Trait Anxiety Inventory (STAI State; total scores 0-80). Trajectories from baseline to post-assessment and follow-up for both TMS groups. Abbreviations: BL, Baseline; Post, Post-assessment; FU, Follow-up.

Figure S8

Average trajectories in means with 95% confidence intervals for the maximal subjective anxiety level (scale from 0 to 100) during the behavioral approach task (BAT) in-vivo. Trajectories from baseline to post-assessment and follow-up for both TMS groups. Abbreviations: BL, Baseline; Post, Post-assessment; FU, Follow-up.

Figure S9

Average trajectories in means with 95% confidence intervals for the maximal distance achieved (from 0 to 11.60 meters) during the behavioral approach task (BAT) in-vivo. Trajectories from baseline to post-assessment and follow-up for both TMS groups. Abbreviations: BL, Baseline; Post, Post-assessment; FU, Follow-up.

**Table S1**

|  |  |  | Skewness | |  | Kurtosis | |  | | Shapiro-Wilk Test | | | | | |
| --- | --- | --- | --- | --- | --- | --- | --- | --- | --- | --- | --- | --- | --- | --- | --- |
| Outcome | TMS group |  | Value | *SE* |  | Value | *SE* |  | | *Test statistic* | | *df* | | *p* | |
| AQ |  |  |  |  |  |  |  |  |  | |  | |  | |  |
| Anxiety | Sham |  | 0.19 | 0.23 |  | -0.59 | 0.45 |  | 0.98 | | 114 | | .229 | |  |
|  | Active |  | 0.08 | 0.23 |  | -0.25 | 0.45 |  | 0.99 | | 113 | | .252 | |  |
| Avoidance | Sham |  | 0.27 | 0.23 |  | -0.95 | 0.45 |  | 0.96 | | 114 | | .002** | |  |
|  | Active |  | 0.58 | 0.23 |  | -0.21 | 0.45 |  | 0.96 | | 112 | | .002** | |  |
| ATHQ | Sham |  | -0.11 | 0.23 |  | -0.57 | 0.45 |  | 0.99 | | 114 | | .333 | |  |
|  | Active |  | -0.13 | 0.23 |  | -0.84 | 0.45 |  | 0.97 | | 113 | | .027* | |  |
| BAT (VR) |  |  |  |  |  |  |  |  |  | |  | |  | |  |
| STAI State | Sham |  | 0.90 | 0.23 |  | 0.57 | 0.45 |  | 0.93 | | 113 | | < .001*** | |  |
|  | Active |  | 0.78 | 0.23 |  | 0.02 | 0.47 |  | 0.94 | | 106 | | < .001*** | |  |
| Max. Distance | Sham |  | -0.07 | 0.23 |  | -1.81 | 0.45 |  | 0.79 | | 113 | | < .001*** | |  |
|  | Active |  | -0.18 | 0.23 |  | -1.72 | 0.47 |  | 0.79 | | 106 | | < .001*** | |  |
| Max. Anxiety | Sham |  | -0.24 | 0.23 |  | -0.97 | 0.45 |  | 0.96 | | 113 | | .001** | |  |
|  | Active |  | -0.34 | 0.23 |  | -1.00 | 0.47 |  | 0.94 | | 106 | | < .001*** | |  |
| BAT (in-vivo) |  |  |  |  |  |  |  |  |  | |  | |  | |  |
| STAI State | Sham |  | 1.05 | 0.23 |  | 0.37 | 0.46 |  | 0.90 | | 111 | | < .001*** | |  |
|  | Active |  | 0.95 | 0.23 |  | 0.13 | 0.47 |  | 0.91 | | 106 | | < .001*** | |  |
| Max. Distance | Sham |  | -1.24 | 0.23 |  | 0.20 | 0.45 |  | 0.71 | | 112 | | < .001*** | |  |
|  | Active |  | -1.43 | 0.23 |  | 0.54 | 0.47 |  | 0.62 | | 106 | | < .001*** | |  |
| Max. Anxiety | Sham |  | 0.17 | 0.23 |  | -1.21 | 0.45 |  | 0.94 | | 112 | | < .001*** | |  |
|  | Active |  | 0.04 | 0.24 |  | -1.24 | 0.47 |  | 0.94 | | 105 | | < .001*** | |  |
| ADS-K | Sham |  | 0.71 | 0.23 |  | -0.29 | 0.45 |  | 0.93 | | 114 | | < .001*** | |  |
|  | Active |  | 0.93 | 0.23 |  | 0.80 | 0.45 |  | 0.93 | | 113 | | < .001*** | |  |
| ASI-3 | Sham |  | 1.07 | 0.23 |  | 1.93 | 0.45 |  | 0.94 | | 114 | | < .001*** | |  |
|  | Active |  | 0.44 | 0.23 |  | -0.75 | 0.45 |  | 0.96 | | 113 | | .001** | |  |

|  |  |  | Skewness | |  | Kurtosis | |  | | Shapiro-Wilk Test | | | | | |
| --- | --- | --- | --- | --- | --- | --- | --- | --- | --- | --- | --- | --- | --- | --- | --- |
| Outcome | TMS group |  | Value | *SE* |  | Value | *SE* |  | | *Test statistic* | | *df* | | *p* | |
| Age | Sham |  | -0.09 | 0.38 |  | -1.09 | 0.75 |  | 0.95 | | 38 | | .073 | |  |
|  | Active |  | 0.49 | 0.38 |  | -0.61 | 0.75 |  | 0.95 | | 38 | | .105 | |  |
| AQ |  |  |  |  |  |  |  |  |  | |  | |  | |  |
| Anxiety | Sham |  | 0.08 | 0.38 |  | -0.80 | 0.75 |  | 0.97 | | 38 | | .428 | |  |
|  | Active |  | 0.53 | 0.38 |  | -0.62 | 0.75 |  | 0.95 | | 38 | | .111 | |  |
| Avoidance | Sham |  | -0.30 | 0.38 |  | -0.05 | 0.75 |  | 0.98 | | 38 | | .731 | |  |
|  | Active |  | 0.36 | 0.39 |  | -0.79 | 0.76 |  | 0.95 | | 37 | | .135 | |  |
| ATHQ | Sham |  | -1.41 | 0.38 |  | 2.33 | 0.75 |  | 0.88 | | 38 | | .001** | |  |
|  | Active |  | -1.32 | 0.38 |  | 1.22 | 0.75 |  | 0.85 | | 38 | | < .001** | |  |
| BAT (VR) |  |  |  |  |  |  |  |  |  | |  | |  | |  |
| STAI State | Sham |  | 0.60 | 0.38 |  | -0.31 | 0.75 |  | 0.95 | | 38 | | .094 | |  |
|  | Active |  | 0.71 | 0.39 |  | 0.24 | 0.77 |  | 0.96 | | 36 | | .161 | |  |
| Max. Distance | Sham |  | 0.88 | 0.38 |  | -0.84 | 0.75 |  | 0.79 | | 38 | | < .001*** | |  |
|  | Active |  | 0.56 | 0.39 |  | -1.44 | 0.77 |  | 0.79 | | 36 | | < .001*** | |  |
| Max. Anxiety | Sham |  | -0.82 | 0.38 |  | 0.56 | 0.75 |  | 0.92 | | 38 | | .009** | |  |
|  | Active |  | -0.60 | 0.39 |  | -0.37 | 0.77 |  | 0.94 | | 36 | | .036* | |  |
| Presence | Sham |  | -0.68 | 0.40 |  | -0.35 | 0.78 |  | 0.93 | | 35 | | .028* | |  |
|  | Active |  | -0.60 | 0.41 |  | -0.77 | 0.81 |  | 0.91 | | 32 | | .012* | |  |
| BAT (in-vivo) |  |  |  |  |  |  |  |  |  | |  | |  | |  |
| STAI State | Sham |  | 0.21 | 0.38 |  | -1.14 | 0.75 |  | 0.95 | | 38 | | .093 | |  |
|  | Active |  | 0.45 | 0.38 |  | -0.86 | 0.75 |  | 0.94 | | 38 | | .052 | |  |
| Max. Distance | Sham |  | -0.35 | 0.38 |  | -1.43 | 0.75 |  | 0.83 | | 38 | | < .001*** | |  |
|  | Active |  | -0.83 | 0.38 |  | -0.74 | 0.75 |  | 0.78 | | 38 | | < .001*** | |  |
| Max. Anxiety | Sham |  | -0.46 | 0.38 |  | -0.84 | 0.75 |  | 0.93 | | 38 | | .019* | |  |
|  | Active |  | -0.60 | 0.39 |  | -0.50 | 0.76 |  | 0.94 | | 37 | | .053 | |  |
| ADS-K | Sham |  | 0.49 | 0.38 |  | -0.92 | 0.75 |  | 0.92 | | 38 | | .013* | |  |
|  | Active |  | 1.45 | 0.38 |  | 2.82 | 0.75 |  | 0.88 | | 38 | | .001** | |  |
| ASI-3 | Sham |  | 0.91 | 0.38 |  | 1.27 | 0.75 |  | 0.95 | | 38 | | .072 | |  |
|  | Active |  | 0.03 | 0.38 |  | -1.18 | 0.75 |  | 0.96 | | 38 | | .139 | |  |
| STAI Trait | Sham |  | 0.47 | 0.38 |  | -0.65 | 0.75 |  | 0.95 | | 38 | | .115 | |  |
|  | Active |  | 0.85 | 0.38 |  | 0.42 | 0.75 |  | 0.94 | | 38 | | .030* | |  |
| TAS | Sham |  | 0.75 | 0.38 |  | 0.06 | 0.75 |  | 0.94 | | 38 | | .033* | |  |
|  | Active |  | 0.90 | 0.38 |  | 0.96 | 0.75 |  | 0.94 | | 38 | | .052 | |  |

**Table S2**

**Table S3**

|  |  |  | Skewness | |  | Kurtosis | |  | | Shapiro-Wilk Test | | | | | |
| --- | --- | --- | --- | --- | --- | --- | --- | --- | --- | --- | --- | --- | --- | --- | --- |
| Outcome | TMS group |  | Value | *SE* |  | Value | *SE* |  | | *Test statistic* | | *df* | | *p* | |
| TMS |  |  |  |  |  |  |  |  |  | |  | |  | |  |
| rMT | Sham |  | 0.47 | 0.38 |  | 0.52 | 0.75 |  | 0.96 | | 38 | | .216 | |  |
|  | Active |  | 0.24 | 0.38 |  | -0.56 | 0.75 |  | 0.98 | | 38 | | .747 | |  |
| TMS intensity | Sham |  | 0.07 | 0.39 |  | -0.39 | 0.76 |  | 0.97 | | 37 | | .377 | |  |
|  | Active |  | 0.14 | 0.38 |  | -0.81 | 0.75 |  | 0.97 | | 38 | | .457 | |  |
| VRET |  |  |  |  |  |  |  |  |  | |  | |  | |  |
| Duration exposure | Sham |  | 1.45 | 0.39 |  | 2.57 | 0.77 |  | 0.88 | | 36 | | .001** | |  |
|  | Active |  | 1.49 | 0.40 |  | 3.13 | 0.79 |  | 0.88 | | 34 | | .002** | |  |
| Duration habituation | Sham |  | 3.42 | 0.38 |  | 12.53 | 0.75 |  | 0.57 | | 38 | | < .001*** | |  |
|  | Active |  | 3.26 | 0.38 |  | 13.38 | 0.75 |  | 0.65 | | 38 | | < .001*** | |  |
| Max. Anxiety | Sham |  | -1.54 | 0.38 |  | 1.44 | 0.75 |  | 0.71 | | 38 | | < .001*** | |  |
|  | Active |  | -1.31 | 0.38 |  | 0.69 | 0.75 |  | 0.74 | | 38 | | .< .001*** | |  |
| Presence | Sham |  | -0.66 | 0.38 |  | 0.10 | 0.75 |  | 0.95 | | 38 | | .119 | |  |
|  | Active |  | -1.04 | 0.38 |  | 0.43 | 0.75 |  | 0.89 | | 38 | | .001** | |  |
| SSQ | Sham |  | 0.77 | 0.39 |  | 0.06 | 0.77 |  | 0.93 | | 36 | | .025* | |  |
|  | Active |  | 1.81 | 0.38 |  | 5.54 | 0.75 |  | 0.86 | | 38 | | < .001*** | |  |
| STAI State | Sham |  | 0.86 | 0.38 |  | 0.03 | 0.75 |  | 0.92 | | 38 | | .012* | |  |
|  | Active |  | 0.41 | 0.38 |  | -0.43 | 0.75 |  | 0.96 | | 38 | | .150 | |  |
| IPQ |  |  |  |  |  |  |  |  |  | |  | |  | |  |
| General Presence | Sham |  | -1.10 | 0.38 |  | 1.35 | 0.75 |  | 0.85 | | 38 | | < .001*** | |  |
|  | Active |  | -1.00 | 0.38 |  | 0.79 | 0.75 |  | 0.87 | | 38 | | < .001*** | |  |
| Spatial Presence | Sham |  | -1.02 | 0.38 |  | 2.47 | 0.75 |  | 0.94 | | 38 | | .041* | |  |
|  | Active |  | -0.26 | 0.38 |  | -0.44 | 0.75 |  | 0.96 | | 38 | | .181 | |  |
| Involvement | Sham |  | 0.15 | 0.38 |  | -1.12 | 0.75 |  | 0.94 | | 38 | | .057 | |  |
|  | Active |  | 0.04 | 0.38 |  | -0.64 | 0.75 |  | 0.96 | | 38 | | .184 | |  |
| Experienced Realism | Sham |  | -0.22 | 0.38 |  | -0.63 | 0.75 |  | 0.95 | | 38 | | .091 | |  |
|  | Active |  | 0.01 | 0.38 |  | -0.15 | 0.75 |  | 0.98 | | 38 | | .677 | |  |

**Table S4**

|  |  |  | Skewness | |  | Kurtosis | |  | | Shapiro-Wilk Test | | | | | |
| --- | --- | --- | --- | --- | --- | --- | --- | --- | --- | --- | --- | --- | --- | --- | --- |
| Outcome | TMS group |  | Value | *SE* |  | Value | *SE* |  | | *Test statistic* | | *df* | | *p* | |
| TMS |  |  |  |  |  |  |  |  |  | |  | |  | |  |
| TMS intensity | Sham |  | 0.27 | 0.39 |  | -0.11 | 0.77 |  | 0.97 | | 36 | | .420 | |  |
|  | Active |  | 0.15 | 0.38 |  | -0.93 | 0.75 |  | 0.97 | | 38 | | .316 | |  |
| VRET |  |  |  |  |  |  |  |  |  | |  | |  | |  |
| Duration exposure | Sham |  | 0.76 | 0.38 |  | -0.18 | 0.75 |  | 0.92 | | 38 | | .012* | |  |
|  | Active |  | 1.21 | 0.38 |  | 1.46 | 0.75 |  | 0.90 | | 38 | | .002** | |  |
| Duration habituation | Sham |  | 1.80 | 0.38 |  | 3.91 | 0.75 |  | 0.83 | | 38 | | < .001*** | |  |
|  | Active |  | 2.07 | 0.39 |  | 4.49 | 0.76 |  | 0.76 | | 37 | | < .001*** | |  |
| Max. Anxiety | Sham |  | -1.22 | 0.38 |  | 0.05 | 0.75 |  | 0.72 | | 38 | | < .001*** | |  |
|  | Active |  | -2.20 | 0.38 |  | 4.67 | 0.75 |  | 0.65 | | 38 | | < .001*** | |  |
| Presence | Sham |  | -0.42 | 0.38 |  | -0.13 | 0.75 |  | 0.96 | | 38 | | .172 | |  |
|  | Active |  | -0.18 | 0.39 |  | -0.76 | 0.76 |  | 0.97 | | 37 | | .349 | |  |
| STAI State | Sham |  | 0.77 | 0.38 |  | 0.12 | 0.75 |  | 0.94 | | 38 | | .053 | |  |
|  | Active |  | 0.67 | 0.38 |  | -0.29 | 0.75 |  | 0.94 | | 38 | | .046* | |  |
| IPQ |  |  |  |  |  |  |  |  |  | |  | |  | |  |
| General Presence | Sham |  | -0.62 | 0.38 |  | -0.05 | 0.75 |  | 0.85 | | 38 | | < .001*** | |  |
|  | Active |  | -0.39 | 0.38 |  | -0.81 | 0.75 |  | 0.89 | | 38 | | .001** | |  |
| Spatial Presence | Sham |  | -0.93 | 0.38 |  | 1.39 | 0.75 |  | 0.95 | | 38 | | .081 | |  |
|  | Active |  | -0.54 | 0.38 |  | 0.46 | 0.75 |  | 0.96 | | 38 | | .199 | |  |
| Involvement | Sham |  | 0.01 | 0.38 |  | -0.75 | 0.75 |  | 0.97 | | 38 | | .299 | |  |
|  | Active |  | -0.01 | 0.38 |  | -0.26 | 0.75 |  | 0.99 | | 38 | | .887 | |  |
| Experienced Realism | Sham |  | -0.12 | 0.38 |  | 0.10 | 0.75 |  | 0.98 | | 38 | | .729 | |  |
|  | Active |  | 0.31 | 0.38 |  | -0.25 | 0.75 |  | 0.97 | | 38 | | .418 | |  |

**Table S5**

|  |  |  | Skewness | |  | Kurtosis | |  | | Shapiro-Wilk Test | | | | | |
| --- | --- | --- | --- | --- | --- | --- | --- | --- | --- | --- | --- | --- | --- | --- | --- |
| Outcome | TMS group |  | Value | *SE* |  | Value | *SE* |  | | *Test statistic* | | *df* | | *p* | |
| Days between sessions |  |  |  |  |  |  |  |  |  | |  | |  | |  |
| First and second TMS-VRET | Sham |  | -0.34 | 0.38 |  | 0.33 | 0.75 |  | 0.95 | | 38 | | .064 | |  |
|  | Active |  | 0.25 | 0.38 |  | 1.59 | 0.75 |  | 0.94 | | 38 | | .040* | |  |
| Second VRET-TMS and Post | Sham |  | 1.68 | 0.38 |  | 4.78 | 0.75 |  | 0.85 | | 38 | | < .001*** | |  |
|  | Active |  | 0.46 | 0.39 |  | 0.61 | 0.77 |  | 0.95 | | 36 | | .093 | |  |
| Post and FU | Sham |  | 1.33 | 0.38 |  | 1.28 | 0.75 |  | 0.86 | | 38 | | < .001*** | |  |
|  | Active |  | 2.14 | 0.40 |  | 5.96 | 0.79 |  | 0.79 | | 34 | | < .001*** | |  |
| First and second TMS-VRET | Sham |  | -0.34 | 0.38 |  | 0.33 | 0.75 |  | 0.95 | | 38 | | .064 | |  |
|  | Active |  | 0.25 | 0.38 |  | 1.59 | 0.75 |  | 0.94 | | 38 | | .040* | |  |
| Frequency of self-exposure | Sham |  | -0.21 | 0.38 |  | -0.77 | 0.75 |  | 0.96 | | 38 | | .179 | |  |
|  | Active |  | -0.65 | 0.39 |  | -0.72 | 0.76 |  | 0.90 | | 37 | | .003** | |  |
| TMS |  |  |  |  |  |  |  |  |  | |  | |  | |  |
| Side Effect - Headaches | Sham |  | 0.37 | 0.62 |  | -0.36 | 1.19 |  | 0.95 | | 13 | | .576 | |  |
|  | Active |  | -0.61 | 0.75 |  | -0.54 | 1.48 |  | 0.90 | | 8 | | .306 | |  |
| Side Effect – Dizziness | Sham |  | 0.82 | 0.85 |  | -1.28 | 1.74 |  | 0.87 | | 6 | | .212 | |  |
|  | Active |  | - | - |  | - | - |  | - | | - | | - | |  |
| Mean TMS intensity | Sham |  | 0.12 | 0.39 |  | -0.45 | 0.76 |  | 0.97 | | 37 | | .485 | |  |
|  | Active |  | 0.15 | 0.38 |  | -0.86 | 0.75 |  | 0.97 | | 38 | | .385 | |  |
| GCA Mean | Sham |  | 1.05 | 0.40 |  | 4.03 | 0.78 |  | 0.91 | | 35 | | .007** | |  |
|  | Active |  | 0.11 | 0.38 |  | -0.22 | 0.75 |  | 0.98 | | 38 | | .695 | |  |
| BL-Post Differences |  |  |  |  |  |  |  |  |  | |  | |  | |  |
| AQ Anxiety | Sham |  | -0.15 | 0.38 |  | -0.61 | 0.75 |  | 0.97 | | 38 | | .475 | |  |
|  | Active |  | -1.22 | 0.38 |  | 1.16 | 0.75 |  | 0.89 | | 38 | | .002** | |  |
| AQ Avoidance | Sham |  | 0.32 | 0.38 |  | -0.31 | 0.75 |  | 0.97 | | 38 | | .283 | |  |
|  | Active |  | -0.55 | 0.39 |  | -0.29 | 0.76 |  | 0.96 | | 37 | | .203 | |  |
| BL-FU Differences |  |  |  |  |  |  |  |  |  | |  | |  | |  |
| AQ Anxiety | Sham |  | -0.19 | 0.38 |  | 0.11 | 0.75 |  | 0.99 | | 38 | | .955 | |  |
|  | Active |  | -0.54 | 0.39 |  | -0.52 | 0.77 |  | 0.95 | | 36 | | .077 | |  |
| AQ Avoidance | Sham |  | 0.00 | 0.38 |  | -0.56 | 0.75 |  | 0.98 | | 38 | | .779 | |  |
|  | Active |  | -0.49 | 0.39 |  | -0.49 | 0.77 |  | 0.96 | | 36 | | .184 | |  |

**Figure S1**


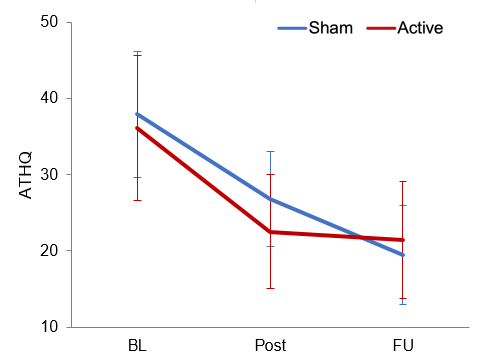


**Figure S2**


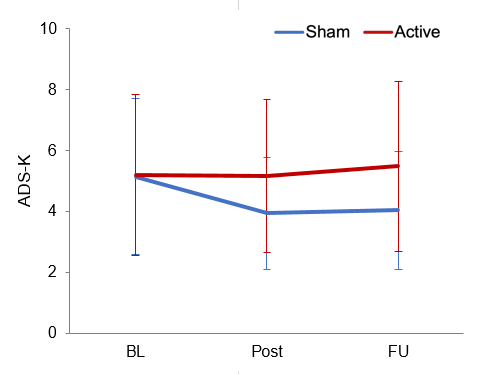


**Figure S3**


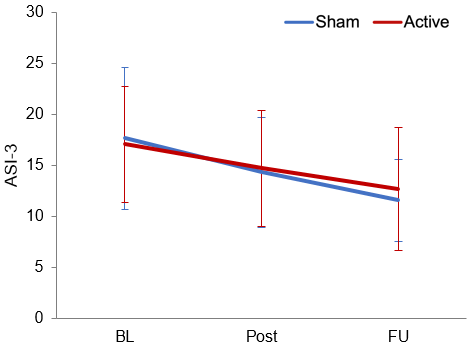


**Figure S4**


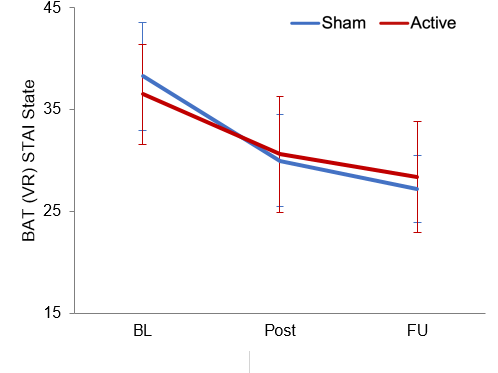


**Figure S5**


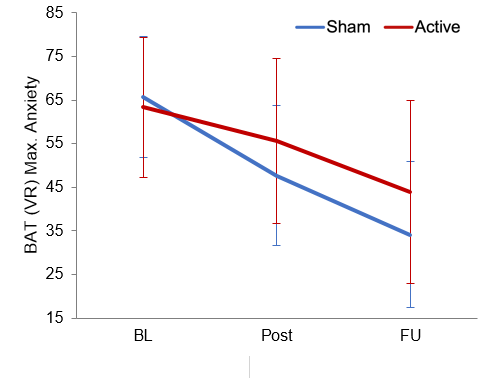


**Figure S6**


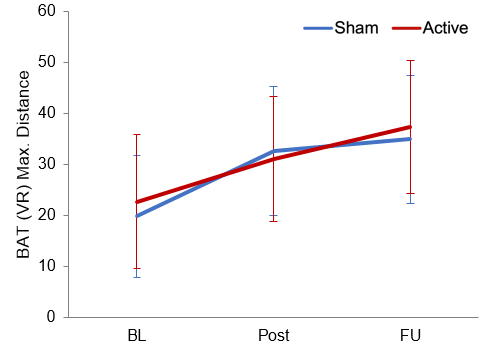


**Figure S7**


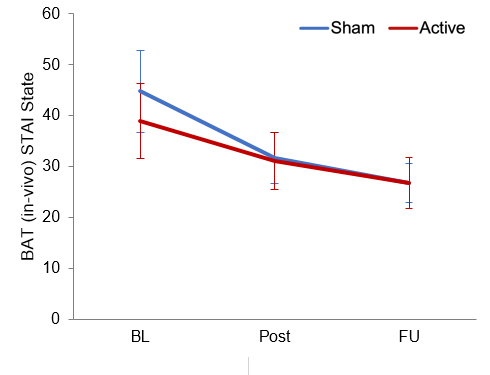


**Figure S8**


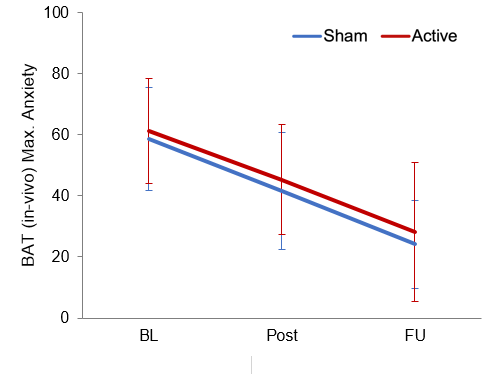


**Figure S9**


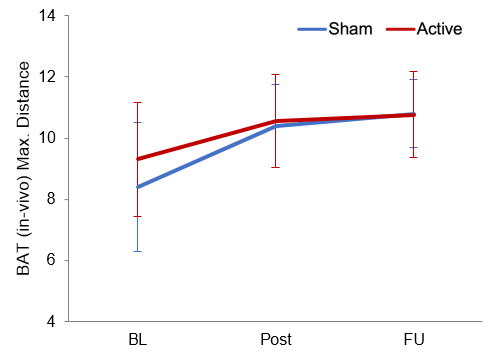

Supplement: Supplementary file 1 — Supplementary Information. [file 41598_2024_80832_MOESM1_ESM.docx]
